# Circulating T-cell phenotypes are influenced by prior *Staphylococcus aureus* exposure in hemodialysis patients

**DOI:** 10.1007/s10096-026-05581-7

**Published:** 2026-07-15

**Authors:** Daniel J. Dempsey, Sultan Imangaliyev, Tracey J. Claxton, Emmett Browne, Roisin Connolly, James Ng, Mark Denton, Brenda Griffin, Mark A. Little, Hilary Humphreys, Arthur White, Rachel M. McLoughlin

**Affiliations:** 1 Host-Pathogen Interactions Group, School of Biochemistry and Immunology, Trinity Biomedical Sciences Institute, Trinity College Dublin, Dublin, Ireland; 2 Department of Clinical Microbiology, Royal College of Surgeons in Ireland University of Medicine and Health Sciences, Dublin, Ireland; 3 Department of Microbiology, Beaumont Hospital, Dublin, Ireland; 4 Trinity Kidney Centre, Trinity Translational Medicine Institute, School of Medicine, Trinity College Dublin, TCD, Dublin, Ireland; 5 Beaumont Kidney Centre, Beaumont Hospital, Dublin, Ireland; 6 School of Computer Science and Statistics, Trinity College Dublin, Dublin, Ireland

**Keywords:** Staphylococcus aureus, Vaccines, T-cell memory

## Abstract

*Staphylococcus aureus* bloodstream infections are a significant risk for hemodialysis patients, who would significantly benefit from a preventative vaccine. To-date *S. aureus* vaccine trials have failed, in part due to lack of consideration of pre-existing immune imprints in relevant patient cohorts. Using a machine learning algorithm this study revealed, prior *S. aureus* exposure in a cohort of 180 hemodialysis patients, was associated with an increase in circulating *S. aureus* antigen-specific exTh17 cells and reduced antigen-specific IL-10 producing CD4 cells and circulating NK cells and Vδ2 cells. It provides novel insights into pre-existing *S. aureus* immunity in hemodialysis patients that could inform next generation vaccine development.

**Supplementary Information:**

The online version contains supplementary material available at https://doi.org/10.1007/s10096-026-05581-7.

## Background

*S. aureus* infections cause ~ 1 million deaths annually [1] and contribute significantly to global antimicrobial resistance burdens [2]. The introduction of an effective vaccine preventing *S. aureus* infection remains a clinical priority. However, despite substantial efforts, an effective anti-*S. aureus* vaccine remains elusive [3], in part due to a lack of identified correlates of immune protection. Exclusive antibody-based vaccination strategies have for the most part been unsuccessful, and the consensus is that next-generation *S. aureus* vaccines, will need to induce protective cellular, in addition to, humoral immunity [4]. Healthy adults possess appreciable numbers of circulating *S. aureus* specific CD4 + T-cells, and it is possible to promote expansion of effector Th1 cells from a pre-existing pool of memory cells upon immunization [5]. However emerging evidence suggests that pre-existing immunity to *S. aureus* can significantly interfere with vaccine efficacy [6, 7], potentially impeding antigen-specific T-cell recall responses [8]. A detailed understanding of pre-existing memory T-cell phenotypes in target patient cohorts is therefore essential for rational vaccine design.

Haemodialysis patients are a target cohort that would benefit significantly from an anti-*S. aureus* vaccine, given their elevated risk for *S. aureus* bloodstream infection. There are however specific challenges associated with inducing effective vaccine-induced immunity in end-stage renal disease patients due to dysfunction in the adaptive immune system [9, 10]. Notably, one of the largest anti-*S. aureus* vaccine trials, undertaken in a haemodialysis patient cohort, failed to reach its desired endpoints despite the induction of robust antibody titres [11]. For haemodialysis patients, the impact of previous *S. aureus* infection, is an essential consideration, given the substantial risk of recurrent infection. However, pre-existing *S. aureus* immunity in haemodialysis patients has not yet been explored.

This study set out to establish if a previous history of *S. aureus* exposure influences circulating leukocyte phenotypes and, importantly, the memory T-cell pool in a haemodialysis patient cohort.

## Methods

The cohort comprises 180 adult haemodialysis patients without active infection, (Supplementary methods for further details). Blood samples were collected from each participant immediately before the haemodialysis procedure and peripheral blood mononuclear cells (PBMCs) isolated within 24 h of for phenotypic analysis. Relevant clinical data was obtained from electronic patient records and local laboratory information systems. Data on previous *S. aureus* isolation from any site were obtained from the clinical microbiology laboratory information system; this included documented positive bloodstream infections and the isolation of *S. aureus* from sites other than the blood. Haemodialysis patients were divided into those with clinically documented previous exposure to *S. aureus* versus no evidence of exposure. Multi-parameter flow cytometry was used to phenotype circulating leukocyte populations and *S. aureus* antigen-specific CD4 + T-cells (Supplementary methods, Supplementary Table S1 & S2, Supplementary Figure S1 & S2). Following data processing, a machine learning algorithm, XGBoost, was used to identify features predictive of previous exposure to *S. aureus* (Supplementary methods for further details).

## Results

Haemodialysis patients (*n* = 180) without any clinical signs of infection were divided into two groups based on previous *S. aureus* exposure (Table 1). Of the 84 participants with clinically documented previous exposure, most (~ 85%) had exposure documented > 12 months before recruitment. As expected, time on dialysis was greater for those with previous *S. aureus* exposure versus those without (mean duration of 54.5 vs. 32.5 months respectively). Previous transplantation was weakly associated with previous *S. aureus* exposure. However, use of immunosuppressive therapy was not statistically significantly associated with previous exposure. Patients with tunneled venous catheters (55 patients) had a slightly higher incidence of previous exposure to *S. aureus* than those with arteriovenous fistula or grafts (19 patients), though the difference did not reach statistical significance.

After data processing (Supplementary methods), 37 features, comprising immune cell phenotypes (Supplementary Tables S1 & S2) and clinical parameters (Table 1), were captured for each patient. This study aimed to identify a set of features among the dataset that are predictive of previous exposure to *S. aureus*. Given the complexity and level of interactivity that was projected to occur with features, an eXtreme Gradient Boost (XGBoost) model [12] was employed. The benefit of XGBoost is that in addition to its flexibility, it contains a suite of regularisation options specifically for the purpose of limiting the degree of overfitting which can occur with other models such as random forest. Once the model was fitted, variable importance was quantified using Shapley values [13] which measure the impact each feature had on the final prediction. When building predictive models, it is standard practice to partition the data into a training set used to fit the model, and a test set used to measure accuracy of the fit. However, preliminary analysis suggested that the model fit was sensitive to the choice of partition. Therefore, to ensure the robustness of conclusions due to the variability in training/test partition results, 1000 randomly selected training/test partitions were selected to fit the data. Aggregated Receiver Operating Characteristic (ROC) curves across all training-test splits returns a test-set AUC of 0.56 (CI: 0.554–0.565), (Supplementary Figure S3) which indicates a relatively weak model performance. Despite this, Shapley values from each partition were aggregated to provide stable rankings of feature importances, with consistently high-ranked features identified as potential predictors.

**Table 1:** Study participant demographics

	No previous exposure	Previous exposure	*p*-values
	*n* = 96	*n* = 84	
Age (mean years ±SE)	59.8±1.7 (*n* = 96)	60.1±1.7 (*n* = 84)	0.9
20-45	35.9±1.3 (*n* = 22)	35.5±2.2 (*n* = 16)	0.89
46-70	59.3±1.1 (*n* = 46)	60±1.2 (*n* = 43)	0.64
>70	79.4±1.2 (*n* = 28)	76±0.8 (*n* = 25)	0.02
Gender			
Female	23.3% (*n* = 42)	15.6% (*n* = 28)	0.2
Male	30.0% (*n* = 54)	31.1% (*n* = 56)	
Time since previous exposure (number of patients)			
< 12 months		4	
> 12 months		71	
Not recorded		9	
Time on dialysis (Mean months ± SE)	30.1 ± 3.8	50.1 ± 5	<0.01
1-48 months	18.4 ± 1.3	19.8 ± 1.7	0.52
49-96 months	65.9 ± 4.6	68.9 ± 2.4	0.58
>96 months	143.2 ± 24.1	141.8 ± 8.5	0.96
Aetiology of CKD (number of patients)			
Chronic glomerulonephritis	16	17	
Ischaemic nephropathy	21	15	
Polycystic kidney disease	12	2	
Diabetes mellitus	26	32	0.09
Congenital	9	9	
Other	10	5	
Unknown	3	6	
Access type (number of patients)			
T-CVC	49	54	0.19
AVF	44	28	
AVG	2	1	
Unknown	1	1	
Immunosuppressive therapy (number of patients)			
No	87	72	0.31
Yes	8	12	
Unknown	1	0	
Previous transplant (number of patients)			
No	86	64	0.04
Yes	10	19	
Unknown	0	1	

Demographic data for 180 recruited patients, for whom systemic immune profiling was completed, are displayed in the table.  Exposure was defined as clinically documented isolation of *S. aureus* from the blood, or a site other than blood e.g. nasal swab or wound swab. Age was calculated as mean years for each grouping plus/minus one standard error (SE). Time on dialysis was calculated as mean months for each grouping plus/minus one standard error (SE). *CKD,* Chronic Kidney Disease; *T-CVC*, Tunneled Central Venous Catheter; *AVF*, Arteriovenous Fistula; *AVG*, Arteriovenous Graft. Immunosuppressive therapy was treatment with Prednisolone, Tacrolimus, Mycophenolate, Lenalidomide, Azathioprine, Cyclophosphamide, Cyclosporine, or Rituximab either alone or up to three medications in combination

*P*-values for numerical demographic features are from t-tests of zero difference, and *p*-values for categorical demographic features are from chi-squared tests of equal proportions across all levels of the variable. Note that the sub-demographic counts may not add to the totals (96 and 84) in some cases, as some patients fell within multiple groups simultaneously, for example, when more than one cause of CKD was identified

**Fig. 1:**
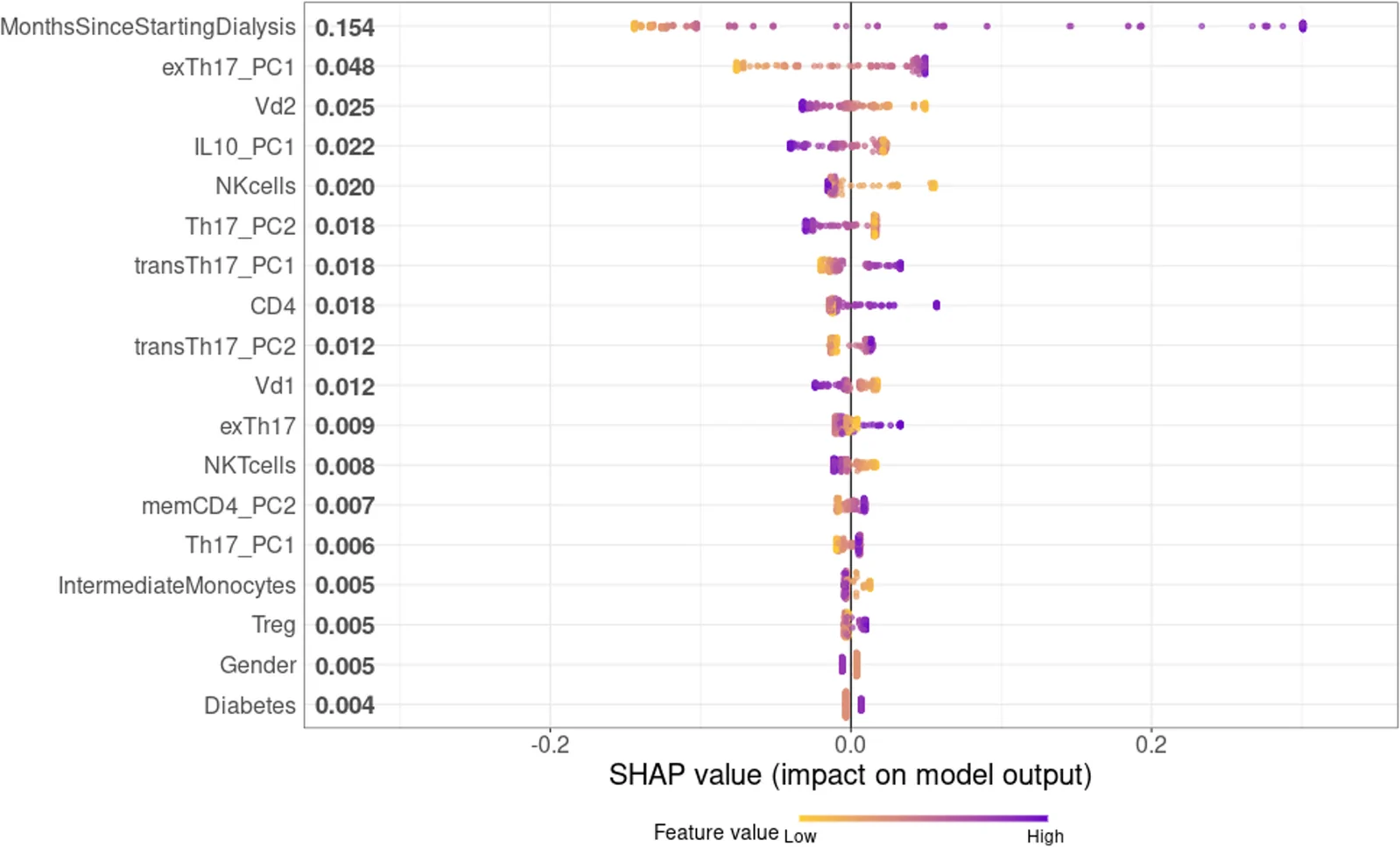
Geometric median of Shapley values for the features most predictive of previous *S. aureus* exposure in a haemodialysis cohort. Data from the 180 patients were partitioned into 1000 randomly partitioned training/test sets and an XGBoost model fitted to determine what features differ between patients with and without previous *S. aureus* exposure. For each fit, Shapley values were computed to measure feature importance and the geometric median of the Shapley values across the 1000 runs is displayed. The colour coding indicates directionality with increased levels of a particular patient’s value donated in purple and reduced levels in yellow. The numbers along the left-hand margin denote the mean of the absolute geometric median Shapley values. A large Shapley value means the feature strongly influences the prediction towards previous exposure vs. no previous exposure. The features are ranked in descending order based on this metric. Only features that performed better than the artificial random noise variable are displayed

Consistent with previous studies [14], the number of months on dialysis was consistently the highest ranked feature associated with previous exposure to *S. aureus* across all 1000 partition runs (Fig. 1). The next top four most important features associated with previous *S. aureus* exposure were distinct T-cell populations. The first principal component (PC1) for *S. aureus* antigen-specific exTh17 cells ranked in the top 10 in 62% of the simulation runs, and PC1 for *S. aureus* antigen-specific IL10 + CD4 cells, along with circulating Vδ2 cells and NK cells, ranked in the top 10 > 40% of the time. It has previously been shown that *S. aureus* antigen specific T-cell responses are not significantly influenced by strain heterogeneity [15] reflecting the fact that most potential T-cell epitopes are likely conserved across strains. Therefore, to account for the high-correlation of antigen-specific T cell responses between strains tested in this study (Supplementary Fig. 4), principial component analysis was employed to reduce dimensionality (Supplementary methods for details).

For months on dialysis, the longer a patient had been on dialysis the greater the Shapley value, indicating a higher predicted probability of previous exposure to *S. aureus*. Higher Shapley values for *S. aureus* antigen-specific exTh17 cells, and lower Shapley values for antigen-specific IL10 + CD4 cells, along with lower circulating Vδ2, NK were also associated with a higher predicted probability of previous *S. aureus* exposure in these patients (Fig. 1).

## Discussion

Although the predictive performance of the explainable machine learning model was limited, this study does suggest that circulating T-cell phenotypes are influenced by previous microbial exposure in a haemodialysis cohort. The model confirmed, that as expected, duration of dialysis is strongly associated with previous *S. aureus* exposure, however, a distinct T-cell signature was also identified to associate with previous *S. aureus* exposure. Importantly when months on dialysis was removed from the analysis, the distinct T-cell signature remained associated with previous exposure (Supplemental Fig. 5). For most patients the exposure to *S. aureus* was documented at least 1 year previously, indicating that these unique signatures are retained for extended periods post clearance of the bacterium.

XGBoost is a powerful non-parametric tool that can fit complex associations, however ultimately the test set performance here was weak, as was the performance of other methods we applied (random forests, Bayesian regression). This is likely due to the limited sample size which impacts the generalizability of findings and limits the predictive capacity of the model. Larger, more diverse datasets would be beneficial for validating and extending these results. However, a significantly limitation of the approach is the challenge faced with defining “previous exposure”. This study categorised patients based on a defined clinical history of *S. aureus* infection which included both bloodstream infection and detection of *S. aureus* from routine clinical screening, however this does not consider patients who had been exposed to *S. aureus* but did not meet the clinical definition employed here. Moreover, the study does not capture exposure to *S. aureus* as a consequence of asymptomatic colonisation. Given that many individuals can be colonised with *S. aureus* from birth and that this colonisation can be transient, it is extremely challenging to establish the impact of asymptomatic *S. aureus* colonisation on host immunity [7]. Employing a similar model to identify features associated with an active *S. aureus* infection in these patients may provide more definitive insights, while further longitudinal studies would be necessary to establish definitive causality. Notwithstanding these limitations, this study provides a useful framework for the design of future studies that are essential to reveal the impact of existing *S. aureus* immune imprints on subsequent responses to immunization and infection.

The study provides some intriguing insights into how *S. aureus* exposure can potentially shape adaptive immunity. *S. aureus* antigen-specific exTh17 cells were expanded in the circulation of previously infected patients. These cells produce IFN-γ, but simultaneously express the Th17 lineage marker CD161 and are considered nonclassical Th1 cells originating from the phenotypic shift of Th17 cells in response to environmental cues. While Th1 cells have previously been shown to be expanded during *S. aureus* bloodstream infection [15], this study reveals that antigen-specific memory cells retained long-term after exposure to *S. aureus* are not classic memory Th1 cells, but rather plastic cells, which potentially retain the ability to respond to subsequent antigen encounter with both the production of IFN-γ and IL-17. Consistent with this, *S. aureus* antigen-specific transitioning Th17 cells (ranked 7th, Fig. 1), which produced both IFN-γ and IL-17 in response to *S. aureus* antigen stimulation, were also expanded in patients with previous *S. aureus* exposure. In a murine model of *S. aureus* sepsis, the trans-differentiation of Th17 cells to Th1 cells was essential to control bacterial burden [16], supporting the importance of polyfunctional T-cells in the protective immune response to *S. aureus*. Previous *S*. *aureus* exposure was also associated with a reduction in IL-10 producing CD4 cells in this cohort, again suggesting CD4 + memory cells that are retained after encountering *S*. *aureus*, may be primed for a more enhanced effector response upon subsequent antigen exposure. Further fate mapping experiments would be required to definitively profile the ontogeny of these *S. aureus* experienced memory T-cells.

Reduced proportions of circulating Vδ2 + γδT-cells and NK cells were also associated with previous *S. aureus* exposure, suggesting that impairments in these innate and unconventional lymphocytes, which is known to occur during ESRD [17, 18], may represent a potential risk factor for *S. aureus* infection in haemodialysis patients. γδT-cells have been identified as important correlates of immune protection during *S. aureus* infection in mice [19], with some evidence that NK cells may also be protective in murine models [20].

Overall, this study supports the ascertain that previous exposure to *S. aureus* can shape CD4 + T-cell memory, resulting in the retention of circulating polyfunctional memory T-cells, and supports the development of novel immunization strategies that could potentially harness the protective effector functions of these cells. Additionally, this study reveals that an impairment in innate and unconventional T-cells may represent an important risk factor for *S. aureus* bloodstream infection, warranting further investigation into the role played by NK and Vδ2 cells in *S. aureus* infection in humans. Most importantly however, it reveals that a history of previous exposure to *S. aureus* significantly shapes a patient’s immunological phenotype, and supports the assertion that this pre-existing immunity is something that must be considered for future *S. aureus* vaccine development [7]. This study points towards the existence of a sustained T-cell imprint in patients that have previously encountered this organism in a pathogenic setting, which could potentially influence a patients’ response to vaccination. Disparity in the pre-existing immunological landscape among haemodialysis patients, due to their prior infection history and potentially the type of prior exposure to *S. aureus* that has been encountered, may in part explain previous vaccine failures in similar cohorts [6].

## Supplementary Information

Below is the link to the electronic supplementary material.

Supplementary Material 1

Supplementary Material 2

## Data Availability

Clinical data can not be publicly shared. All code is available through the following link to Github: https://github.com/DanDempsey/S_Aureus_EML_Code.
