# Supplementary material for: Circulating T-cell phenotypes are influenced by prior Staphylococcus aureus exposure in hemodialysis patients
Source: Eur J Clin Microbiol Infect Dis. 2026 Jul 15;45(10):3269–74. doi: 10.1007/s10096-026-05581-7 (PMC13676517; doi:10.1007/s10096-026-05581-7)
Supplement: Supplementary file 1 [file 10096_2026_5581_MOESM1_ESM.docx]

**Supplementary Tables and Figures**

**Table S1: Characterisation of circulating innate and T cell populations using surface expressed markers**

| **Cell type** | **Antigen expression** |
| --- | --- |
| **Innate cell subsets:**  NK cells  NK T cells  B cells  Classical monocytes  Intermediate monocytes  Non-classical monocytes  Total T cells | CD56+CD3-  CD56+CD3+  CD19+CD3-  CD14+CD16-  CD14+CD16+  CD14-CD16+CD56-CD8-  CD3+ |
| **Ɣδ T cell subsets:**  Vδ1 | CD3^+^Vδ1^+^Vδ2^-^  CD3^+^Vδ1^-^Vδ2^+^ |
| Vδ2 |  |
| **αβT cell subsets:**  CD8+ | CD3^+^CD4^-^CD8^+^  CD3^+^CD4^+^CD8^-^  CD3^+^CD4^+^CD25^+^CD127^low/-^  CD3^+^CD4^+^CD25^-^CD161^-^CXCR3^+^  CD3^+^CD4^+^CD25^-^CD161^-^CXCR3^-^CCR6^-^CCR4^+^  CD3^+^CD4^+^CD25^-^CD161^+^CXCR3^-^CCR6^+^  CD3^+^CD4^+^CD25^-^CD161^+^CXCR3^+^ |
| CD4^+^ |  |
| Activated Treg |  |
| Th1 |  |
| Th2 |  |
| Th17  exTh17 |  |
|  |  |

“+” suffix:	indicates positive expression of a specified cell surface marker

“-“ suffix:	indicates negative expression of a specified cell surface marker

“low” suffix:	indicates low expression

“NK”	indicates Natural Killer cells

“Treg”:	regulatory T cell

**Table S2: Identification of *S. aureus*-antigen specific memory T cell responses characterized using surface markers and intracellular cytokines**

| **Cell type** | **Antigen expression** |
| --- | --- |
| CD4 T cells  Memory T cells  Proliferating memory T cells  Proliferating memory IL-10 producers  Proliferating memory Th1  Proliferating memory Th17  Proliferating memory TransTh17  Proliferating memory exTh17 | CD4^+^  CD4^+^CD45RO^+^  CD4^+^CD45RO^+^CFSE^lo^  CD4^+^CD45RO^+^CFSE^lo^IL10^+^  CD4^+^CD45RO^+^CFSE^lo^IFNɣ^+^IL17AF^-^  CD4^+^CD45RO^+^CFSE^lo^IFNɣ ^-^IL17AF^+^  CD4^+^CD45RO^+^CFSE^lo^IFNɣ ^+^IL17AF^+^  CD4^+^CD45RO^+^CFSE^lo^IFNɣ ^+^CD161^-^ |

“+” suffix:	indicates positive expression of a specified cell surface marker

“-“ suffix:	indicates negative expression of a specified cell surface marker

“lo” suffix:	indicates low expression

“TransTh17”	indicates transitioning Th17 cells

**Table S3: XGBoost model tuning parameters, their interpretation, and the grid points utilised in the 10-fold cross-validation**

| **Tuning parameter** | **Grid points** | **Explanation** |
| --- | --- | --- |
| Learning rate | 0.005, 0.01, 0.05, 0.1 | A weight applied to each component tree when calculating its contribution to correcting the residual error. This protects against a relatively simple tree from dominating the model, which in turn can inhibit the exploration of how other combinations of features may contribute to the predictive accuracy. |
| Maximum tree depth | 1, 3, 5 | The maximum allowed depth of each tree. Prevents any one tree from growing too complex and overfitting. |
| Lagrange multiplier | 5, 10, 15 | A lower bound on how much the tree must reduce the error before a proposed split is accepted. Therefore the tree will not grow more complex only to correct very minor (and probably irrelevant) sources of error. |
| L2 Penalty parameter | 0.1, 0.9 | Similar to the learning rate, this prevents any component tree from dominating its contribution to the error reduction. It does so by utilising a combined penalty across all the component trees, instead of “one at a time” like the learning rate. |
| Subsampling ratio | 0.5, 0.75, 1 (rows)  0.5, 0.75, 1 (columns) | Each tree is only built on a random sample of the data (both rows and columns), and this parameter sets the proportion of data that we use. This prevents certain data points (such as outliers) from overly influencing the model fits. |
| Number of component trees | 10, 20, 50, 100 | This is set in conjunction with the learning rate; a smaller learning rate implies we need more component trees (and vice-versa) since each tree is given less weight. |

Underlined numbers denote the values that were chosen by the cross-validation procedure. The cross-entropy statistic was used as the cross-validation optimisation metric. Any parameter not listed here was kept to the default value as implemented by the *xgboost* package in R^S8^.

**Figure S1**

**A**

**B**

**Figure S1: Gating Strategy for phenotypic analysis of circulating PMBC populations**

PMBC were collected from each patient and circulating leukocyte populations assessed by flow cytometry using two separate antibody panels. Gating was as follows on the innate cell panel (A) total cells were gated on single cells. NK cells were identified as CD56+ which were then sub-divided into CD3+ or CD3-. CD14 v CD16 cells were gated on the single cell population to determine classical monocytes (CD14+CD16-) and intermediate monocytes (CD14+CD16+). The CD14-/loCD16+ population was then gated on CD56 and CD3 to identify non-classical monocytes (CD14-/loCD16+CD56-CD3-). CD19 v CD3 cells were gated on the single cell population to determine the percentage of B cells (CD19+CD3-). The CD3+ population was then gated on CD8+ cells (CD3+CD8) or on Vδ1 and Vδ2 cells to identify γδ T cell populations (CD3+Vδ1+Vδ2- (termed Vδ1 cells) and the CD3+Vδ1-Vδ2+ (termed Vδ2 cells) populations). A second panel was used to further assess CD4+ T cell populations (B). Total cells were gated on single cells. CD3+ cells were determined as a percentage of the single cell population with CD4+ cells being determined as a percentage of the CD3+ population. The CD4+ population was gated on CD25 and CD127 cells to determine the percentages of T-regs (CD25+CD127lo) and effector T cells (CD25-). Effector T cells were then gated on CD161 and CXCR3 cells to determine the percentage of Th1 cells (CD161-CXCR3+), exTh17 cells (CD161+CXCR3+), Th17 cells (CD161+CXCR3-). The CD161-CXCR3- cells within this plot were gated on with CCR4 and CCR6 cells to determine Th2 cells (CCR4+CCR6-).

**Figure S2**

**Figure S2: Gating Strategy for *S. aureus* antigen specific T-cell phenotyping**

PMBC were collected from each patient, CFSE labelled and incubated in vitro with HK-*S. aureus* before assessment of *S. aureus* antigen-specific T-cell responses on day 8 by flow cytometry. Total cells were gated on (FSC-S v SSC-S) to remove debris and (FSC-A v FSC-W) to remove any doublets. The proportions of live cells present within the single cell was determined as FixVia-eF506- cells. Live cells were then gated on CD4+CD45RO+ to identify memory CD4 cells. Memory cells were then gated on CFSE to identify dividing Memory cells (CD4+CD45RO+CFSElo). Cytokine production by the dividing memory cells was then assessed to identify exTh17 cells (IFNγ+CD161+) Th1 cells (IFNγ+IL17AF-), transitioning Th17 cells (IFNγ+IL17AF+) Th17 cells (IFNγ-IL17AF+) and IL10+ CD4 cells (CD4+CD45RO+CFSEloIL-10+). Unstimulated cells were used to determine the percentage of cell proliferation and cytokine production in the sample without any stimulation, and was subsequently subtracted from all antigen stimulated samples from the same individual to determine the antigen specific CD4+ T cell responses.

**Figure S3**

**Figure S3: Aggregated ROC curves across the 1000 training/test set patrician runs.**

Data from the 180 patients was partitioned into 1000 randomly partitioned training/test sets and an XGBoost model fitted to determine what features differ between patients with and without prior *S. aureus* exposure. For each fit, Shapley values were computed to measure feature importance. The above graph shows the ROC curves of model fits. The bands represent the interquartile ranges of the ROC curves, and the solid line is the median. Training set is represented in blue and the test set is represented in orange. The mean AUC is 0.764 (CI: 0.762-0.766) and 0.560 (CI: 0.554-0.565) for the training and test curves respectively.

**Figure S4**

**Figure S4: Correlation matrix of the pre-processed data.**

This graph displays the correlation between each feature in the raw data. The only form of processing applied to the data given here is that the categorical features (gender, aetiology of CKD, etc.) were one-hot encoded (i.e., converted to numerical data via the use of dummy variables). Also included here is a synthetic random noise variable (labelled “random_num”) for validation purposes when fitting the model. The main diagonal is measuring each feature’s correlation with itself, which is always equal to 1. Notice the visible “clusters” along the main diagonal, which represent highly correlated antigen-specific responses to the three individual strains of *S. aureus* used in the in vitro assay.

**Figure S5**

**Figure S5: Proportion of variance explained by the principal components for the *S. aureus* antigen specific response elicited in response to three individual *S. aureus* strains**

To address the high-correlation clusters between antigen specific responses, for proliferating memory CD4+ cells, IL-10 producing memory CD4+ cells, memory Th17, memory Transitioning Th17, memory Th1, memory exTh17 cells, the raw values for the response to an individual strain were replaced by their principal components. Memory CD4 and proliferating Memory CD4 are combined as both groups were highly correlated with each other. To reduce the predictor set, only enough principal components to explain at least 80% of the variance of the original observations were retained. In all cases this resulted in retention of no more than two principal components. The blue bars denote the principal components that were retained for use in the model.

**Figure S6**

**Figure S6: Geometric median of Shapley values for the features most predictive of previous *S. aureus* exposure in a haemodialysis cohort when months on dialysis is removed.**

Data from the 180 patients were partitioned into 1000 randomly partitioned training/test sets and an XGBoost model fitted to determine what features differ between patients with and without previous *S. aureus* exposure. For each fit, Shapley values were computed to measure feature importance and the geometric median of the Shapley values across the 1000 runs is displayed for all features excluding months on dialysis. The colour coding indicates directionality with increased levels of a particular patient’s value donated in purple and reduced levels in yellow. The numbers along the left-hand margin denote the mean of the absolute geometric median Shapley values. A large Shapley value means the feature strongly influences the prediction towards previous exposure vs. no previous exposure. The features are ranked in descending order based on this metric. Only features that performed better than the random noise variable are displayed.

**Figure S7**

**Figure S7: Distribution of Shapley Value Rankings**

Data from the 180 patients was partitioned into 1000 randomly partitioned training/test sets and an XGBoost model fitted to determine what features differ between patients with and without prior *S. aureus* infection. For each fit, Shapley values were computed to measure feature importance. The above graph displays the distribution of the ranks of these Shapley values across all the 1000 partition runs. Only features that were greater than the artificial random noise in terms of the mean of the absolute geometric median Shapley values are displayed here. Most distributions show bi-modality; i.e. the ranks were either very high or very low depending on the specific partition. The top ranked features rank highly more often than the lower ranked features.

**Figure S8**

**Figure S8: Violin plots of the raw data for the top five ranked features**

Raw data for the number of months on dialysis, first principal components (PC1) reflecting a linear combination of the proportions of the raw data for proportions of exTh17, IL-10 producing CD4+ cells, and proportions Vδ2+ and NK cells were plotted for patients with documented prior exposure to *S. aureus* (n=84) versus those with no documented prior exposure (n=96).
