# Supplementary material for: Circulating T-cell phenotypes are influenced by prior Staphylococcus aureus exposure in hemodialysis patients
Source: Eur J Clin Microbiol Infect Dis. 2026 Jul 15;45(10):3269–74. doi: 10.1007/s10096-026-05581-7 (PMC13676517; doi:10.1007/s10096-026-05581-7)
Supplement: Supplementary file 2 [file 10096_2026_5581_MOESM2_ESM.docx]

**Supplementary Methods**

**Collection and processing of patient samples**

This study was approved by the Beaumont Hospital Ethics Committee and the St James’s/Tallaght University Hospital Ethics Committee. The cohort comprises 180 adult haemodialysis patients without active infection, attending Beaumont and Tallaght University Hospitals in Dublin between February 2017 and January 2020. Exclusion criteria were: (1) haemoglobin <10 g/dL or evidence of erythropoietin resistance as defined by epoetin theta doses >8000 IU 3 times weekly, (2) known active infection with blood-borne viruses and (3) current psychiatric inpatients. All participants provided written informed consent. Most patients recruited to the study were dialysed using high-flux synthetic biocompatible membranes [FX CorDiax, Fresenius Medical Care, Germany], with bicarbonate dialysate and water processed in an on-site treatment plant in accordance with international standards.

**Phenotyping of circulating peripheral blood mononuclear cells**
Approximately 30ml of blood was collected from each participant in lithium heparin tubes [Vacuette, Greiner Bio-One International, Austria] immediately before the haemodialysis procedure and peripheral blood mononuclear cells (PBMCs) were isolated within 24 hours of collection by density centrifugation over a Lymphoprep gradient. PBMC were stained with panels of fluorescent monocolonal antibodies against CD3-PerCP-Cy5.5 (Clone OKT3, Invitrogen), CD8-PE-Cγ5 (Clone RPA-T8, Invitrogen), Vδ1-APC (Clone REA173, Miltenyi), Vδ2-BV711 (CloneB6, Biolegend), CD14-BV786 (Clone M5E2, BD), CD16-eF450 (Clone eBioCB16, Invitrogen), CD19-PE-Cγ7 (Clone SJ25C1, BD), and CD56-APC-R700 (Clone NCAM16.2, BD) (innate cell panel) and CD3-PerCP-Cγ5.5 (Clone OKT3, Invitrogen), CD4-PE-Cγ7 (Clone RPA-T4, Invitrogen), CD25-BB515 (Clone 2A3, BD), CD127-APC-eF780 (Clone eBioRDRS, Invitrogen), CD45RO-PE (Clone UCHL1, Invitrogen), CD161-PE-Cγ5 (Clone DX12, BD), CCR4-BV421 (Clone 1G1, BD), CCR6-BV786 (Clone11A9, BD), and CXCR3-PE-CF594 (Clone 1C6/CXCR3, BD) (T-cell panel) and were acquired on the BD LSR Fortessa using Diva software (BD Biosciences) and analysed using FlowJo software (Tree Star, Inc). Fluoresence-minus-one controls were used for gating on leukocyte populations based on their appearance in side scatter and forward scatter (Supplementary Figure S1) and individual cell phenotypes determined (Supplementary Table S1).

**Preparation of heat-inactivated *S. aureus* strains**

*S. aureus* strain PS80 is a clonal complex eight laboratory strain that has been previously described^15^. In addition, this study included 2 representative clinical bloodstream infection isolates that had previously been isolated, which represent dominant circulating methicillin resistant *S. aureus* (MRSA) (cc22) and methicillin-sensitive *S. aureus* (MSSA) (cc7) clones^15^. Bacteria were cultured overnight in Tryptic Soy Broth (TSB) (Fannin). Bacteria were suspended in sterile PBS (Sigma-Aldrich), diluted to 1 x 10^8^ CFU/ml based on optical density measurements, and heat inactivated in a dry block heater at 90°C for 45 min. The suspensions were centrifuged and washed in sterile PBS to remove secreted proteins then resuspended for use. The Pierce Micro BCA Protein Assay Kit (Thermo Fisher Scientific) was used to determine total protein concentration in each stock solution to ensure a standard quantity of protein was added to each assay.

**Assessment of circulating *S. aureus* antigen-specific T-cell responses**
PBMC’s (2 x10^5^ per well) were labelled with CFSE (Invitrogen/Thermo Fisher Scientific) and were cultured in complete RPMI 1640 (Sigma-Aldrich), supplemented with 10% heat-inactivated FBS (Sigma-Aldrich), 2mM L-glutamine, 100U/l penicillin, and 1 mg/ml streptomycin (Sigma-Aldrich) either alone (negative control) or in presence of SEA (Sigma-Aldrich) at a concentration of 1 μg/ml (positive control), or heat-inactivated *S. aureus* strains: PS80, MRSA, MSSA at a concentration of 25μg/ml. Cells were then incubated at 37°C 5% CO_2_. On day 8, phorbol 12-myristate 13-acetate (PMA) (50 ng/mL: Sigma-Aldrich), ionomycin (500 ng/mL; Sigma-Aldrich), and Brefeldin A (5 μg/mL; VWR) were added to the cultures for the final 4 hours. Cells were then harvested by centrifugation before extracellular and intracellular staining. Cells were resuspended in 1:1000 dilution of Fixable Viability Stain (Life Technologies/Thermo Fisher Scientific) for live/dead cell determination. Cells were subsequently resuspended in 10% BSA (Sigma- Aldrich) and stained with fluorochrome-conjugated antibodies against CD4-PE-Cγ7 (Clone RPA-T4, Invitrogen), CD45RO-PE (CloneUCHL1, Invitrogen), CD161-PE-Cγ5 (Clone DX12, BD), IFNγ-APC-eF780 (Clone 4S.B3, Invitrogen), IL17AF-eF450 (Clone 201JS09, Invitrogen), and IL10-eF660 (Clone JES3-9D7, Invitrogen). Cells were analysed by flow cytometry as described above to identify populations of *S. aureus antigen* specific T cells (Supplementary Table S2). Fluorescence-minus-one controls were used for gating cytokine positive cells. Gating strategies are shown in Supplemental Figure S2. Values were corrected for background proliferation by subtracting response to negative unstained cells.

**Data Pre-Processing**

The raw data comprised 180 patients with approx. 42 individual data points. Most of the data was numeric, but some (such as gender, transplant recipient, etc.) were categorical. These were converted to dummy variables via one-hot encoding. In some cases, a patient belonged to more than one category for a given categorical variable. For example, chronic kidney disease (CKD) aetiology where more than one cause was logged. In these cases, the dummy variables were assigned a value of 1 for each category that applied. After one-hot encoding the categorical variables, there were 48 data points for each patient.

Of the 180 patients, only four had missing values and each of those had at most only two missing values. These values were imputed using predictive mean matching^S1^.

To address the high-correlation clusters between antigen-specific T cell responses and other highly correlated cell types (Supplementary Figure S4), Principal Component Analysis (PCA) was employed to reduce dimensionality while retaining essential information. We retained enough principal components to explain at least 80% of the variance in each cluster (Supplementary Figure S6). These principal components were then included in the XG Boost model to represent the combined *S. aureus* antigen specific T-cell response to the three individual strains and feature labels are appended with the relevant principal component (PC) i.e. PC1 or PC2.

The final step of data pre-processing was to include a simulated noise variable, which was generated from a half-normal distribution. This was to set a performance benchmark; any feature that was less predictive than random noise can be safely considered unlikely to have any predictive value at all. Half normal distribution was chosen because, like the actual data, it is skewed, and thus more closely resembles the distribution of the data than a standard normal.

After processing, the final data set had 180 rows (patients) and 37 columns (features or variables), including the artificial random noise variable.

**Machine-Learning Methodology**

The XGBoost model was selected to identify patterns in the data that would account for the complexity and levels of interactivity between the features assessed. Such a high-dimensional data set is likely going to be linearly non-separable, and traditional univariate tests may not be capable of capturing the multivariate interactions between all the different immunological features or account for the complex relationship between the predictors and the response. The XGBoost model also benefits from its flexibility, it contains a suite of regularisation options specifically for the purpose of limiting the degree of overfitting. A random forest model was previously utilized, while it gave consistent results as the XGBoost in terms of variable importance, it led to significant overfitting.

XGBoost requires the user to set several tuning parameters, which control the degree of regularisation. To choose the optimal parameters, a 10-fold cross-validation search across a grid of values was performed (Supplementary Table S3). The grid values were chosen to balance flexibility and regularisation. After tuning parameters were chosen, bootstrapping was used to fit an XGBoost model to 1000 different random training/test set allocations.

Once the models were fitted, variable importance was quantified using Shapley values^S3^ which measure the impact each feature had on the final predictions. Note that Shapley values cannot, on their own, tell you if the model is predictive; it can only be used as a *relative* measure of feature importance^S2, S3^. [The random noise variable was used as the benchmark for feature importance.](https://arxiv.org/pdf/2002.11097)

Supplementary Figure S7 shows the distribution of the Shapley value ranks across bootstrap repetitions; the higher ranked variables tend to have two modes which is a manifestation of the sensitivity of training/test partitions. However, the top ranked features ranked highly more often than lower ranked features. The raw values of the top five ranked features separated by prior infection status are shown for illustrative purposes in Supplementary Figure S8.

**Data and Software Availability**
All data analysis was performed through R version 4.2.1^S4^. The code made use of a number of bespoke packages for R, primarily mice^S5^, xgboost^S6^, SHAPforxgboost^S7^ and ggplot2^S8^ among others. All code is available through the following link to Github: *https://github.com/DanDempsey/S_Aureus_EML_Code*. The data can be made available upon request to the corresponding author.

**Supplementary References**

S1. Rubin, DB. (1986). "Statistical Matching Using File Concatenation with Adjusted Weights and Multiple Imputations". Journal of Business & Economic Statistics. 4 (1): 87–94. DOI 10.2307/1391390. JSTOR 1391390

S2. Fryer D, Strumke A, Nguyen H. (2021). Shapley values for feature selection: The good, the bad, and the Axioms. IEEE Access DOI 10.1109/ACCESS.2021.3119110

S3. Kumar E, Venkatasubramanian S, Scheidegger C, Friedler SA. (2020). Problems with Shapley-value-based explinations as feature importance measures. arXix DOI 10.48550/arXiv.2002.11097

S4. R Core Team (2022). R: A language and environment for statistical computing. R Foundation for Statistical Computing, Vienna, Austria. URL <https://www.R-project.org/>.

S5. van Buuren, S, Groothuis-Oudshoorn, K (2011). mice: Multivariate Imputation by Chained Equations in R. Journal of Statistical Software, 45(3), 1-67. DOI 10.18637/jss.v045.i03.

S6. Chen T, He T, Benesty M, Khotilovich V, Tang Y, Cho H, Chen K, Mitchell R, Cano I, Zhou T, Li M, Xie J, Lin M, Geng Y, Li Y, Yuan J (2024). xgboost: Extreme Gradient Boosting. R package version 1.7.7.1, <https://CRAN.R-project.org/package=xgboost>

S7. Liu Y, Just A (2021). SHAPforxgboost: SHAP Plots for 'XGBoost'. R package version 0.1.1, <https://CRAN.R-project.org/package=SHAPforxgboost>.

S8. H. Wickham (2016). ggplot2: Elegant Graphics for Data Analysis. Springer-Verlag New York. DOI 10.1007/978-3-319-24277-4
